# Human intestinal spheroids cultured using Sacrificial Micromolding as a model system for studying drug transport

**DOI:** 10.1038/s41598-019-46408-0

**Published:** 2019-07-09

**Authors:** Karen E. Samy, Elizabeth S. Levy, Kiet Phong, Benjamin Demaree, Adam R. Abate, Tejal A. Desai

**Affiliations:** 1 Department of Bioengineering and Therapeutic Sciences, University of California, San Francisco, CA USA; 2 UC Berkeley – UCSF Graduate Program in Bioengineering, UCSF Mission Bay Campus, San Francisco, CA USA; 3 Chan Zuckerberg Biohub, San Francisco, CA USA

**Keywords:** Tissue engineering, Drug screening

## Abstract

*In vitro* models of the small intestine are crucial tools for the prediction of drug absorption. The Caco-2 monolayer transwell model has been widely employed to assess drug absorption across the intestine. However, it is now well-established that 3D *in vitro* models capture tissue-specific architecture and interactions with the extracellular matrix and therefore better recapitulate the complex *in vivo* environment. However, these models need to be characterized for barrier properties and changes in gene expression and transporter function. Here, we report that geometrically controlled self-assembling multicellular intestinal Caco-2 spheroids cultured using Sacrificial Micromolding display reproducible intestinal features and functions that are more representative of the *in vivo* small intestine than the widely used 2D transwell model. We show that Caco-2 cell maturation and differentiation into the intestinal epithelial phenotype occur faster in spheroids and that they are viable for a longer period of time. Finally, we were able to invert the polarity of the spheroids by culturing them around Matrigel beads allowing superficial access to the apical membrane and making the model more physiological. This robust and reproducible *in vitro* intestinal model could serve as a valuable system to expedite drug screening as well as to study intestinal transporter function.

## Introduction

*In vitro* models of the small intestine that better recapitulate the *in vivo* epithelial barrier are crucial tools to help predict intestinal uptake of drug candidates before costly and laborious animal studies. The two-dimensional (2D) monolayer transwell model has been widely used to predict drug permeability across the intestinal epithelial barrier. When cultured on the transwell membrane for 21 days, the colon adenocarcinoma cells (Caco-2) form a tight barrier and spontaneously differentiate to display intestinal enterocyte-like characteristics^1^. However, it is now well-established that 2D models suffer many disadvantages including lack of tissue-specific architecture, cell-cell and cell-matrix interactions, as well as biochemical and mechanical cues^2,3^. Such interactions with the tissue microenvironment play an important role in determining cell phenotype and functionality, and consequently affect drug transport and drug responses. Hence, there is a crucial need for new *in vitro* models that can recapitulate the complex functions of the human small intestine while efficiently and reliably predicting human responses.

Three-dimensional (3D) cell culture models in which cells are grown within extracellular matrix (ECM) gels are gaining interest for drug discovery and screening as they are thought to induce expression of more tissue-specific function^4^. Matrix effects have been shown to regulate intestinal epithelial differentiation and proliferation. For example, brush border enzyme expression and activity are significantly stimulated while proliferation is slowed down when Caco-2 cells are cultured on a type I collagen or a laminin matrix compared to tissue culture plastic suggesting that integrin-mediated interactions with basement membrane proteins play a crucial role in guiding the intestinal epithelial phenotype^5^. When cultured in an ECM, Caco-2 cells self-organize into spheroids with a centralized lumen surrounding a monolayer of cells which have the advantage of recreating the spatial organization of intestinal epithelial cells *in vivo*^6^. These spheroids have physiological cell-cell and cell-matrix interactions and are easily scalable and inexpensive. They, thus, offer the potential to serve as an improved platform for drug discovery and screening that can better simulate intestinal epithelial responses to drugs and stimuli compared to the current 2D monolayer model.

Many different methods have been used to culture spheroids including low adhesion plates, hanging drop plates, and micro-patterned scaffolds^7^. We recently developed a novel Sacrificial Micromolding technique to culture geometrically controlled Caco-2 spheroids that are able to lumenize and polarize after only 6 days in culture^6^. However, the characteristics of Caco-2 spheroids including their barrier function and potential changes in cell phenotype and transport function brought about by the 3D microenvironment have not been characterized. Characterization of this model is essential for its application in drug screening as well as studies of epithelial biology. Here, we report that self-assembling multicellular intestinal spheroids cultured using Sacrificial Micromolding display reproducible intestinal features and functions that are more representative of the *in vivo* small intestine. Additionally, we show that cell polarization and maturation occur faster in spheroids than on transwells and that the spheroids are viable for a longer period of time. We also show for the first time the ability to culture Caco-2 cells around Matrigel beads to create inverted spheroids allowing for superficial access to the apical membrane of the cells. Given the simple construction and the ability of the spheroids to reproduce key intestinal features and functions, this system offers the potential to provide readouts that better predict *in vivo* drug permeability than standard 2D cultures while increasing the throughput of drug screening. Moreover, using this model to study the intestinal epithelial barrier could reveal new insights about how therapeutics cross the small intestine *in vivo* and ways in which we can modulate drug transport to increase oral bioavailability of drugs.

## Results

### Culture of lumenized intestinal spheroids using Sacrificial Micromolding

**Figure 1:**
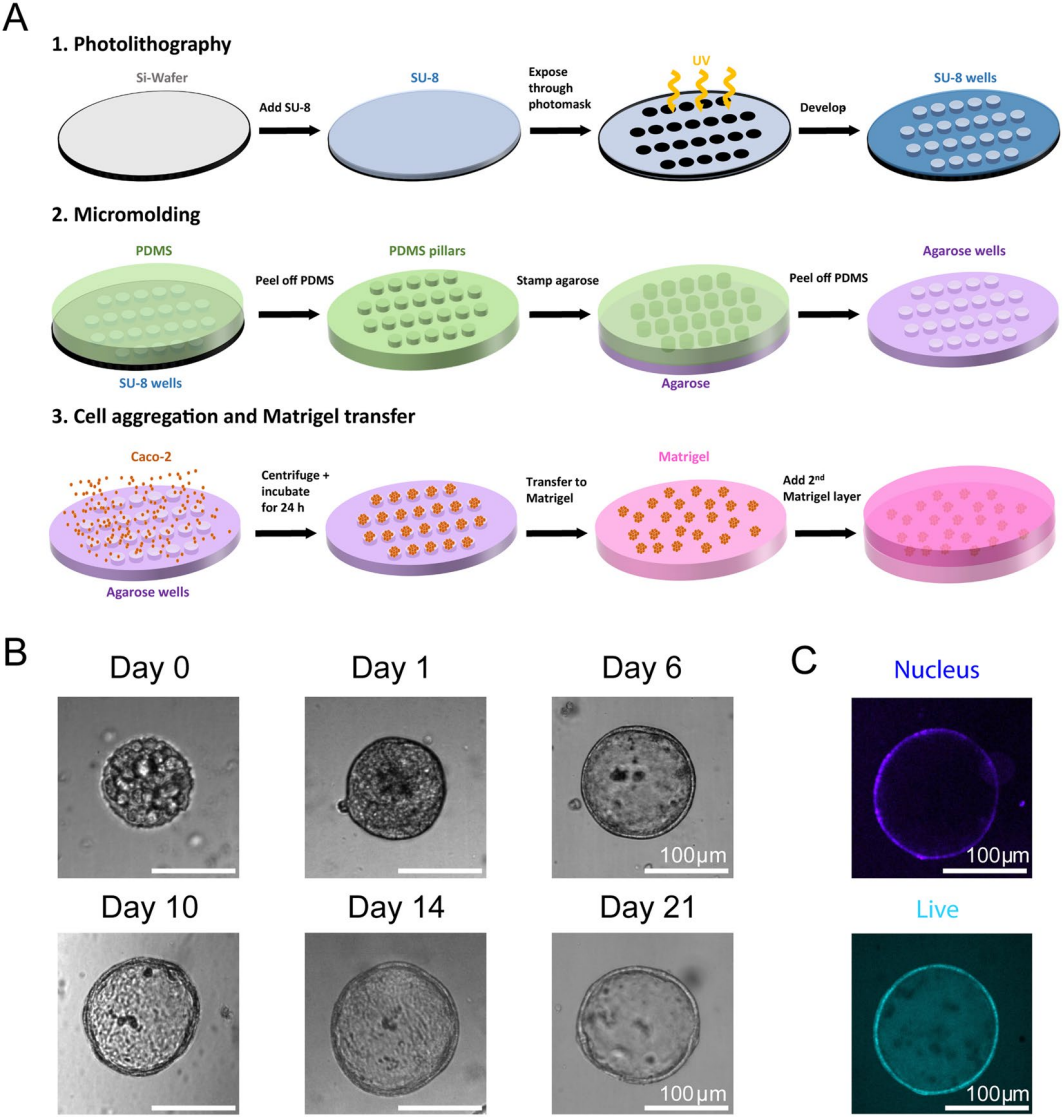
Culture of geometrically-controlled intestinal spheroids using Sacrificial Micromolding. (**A**) Schematic representation of the formation and use of agarose micromolds for patterning Caco-2 spheroids in a soft Matrigel matrix. (**B**) Representative brightfield confocal images (10X) of Caco-2 cells in agarose microwells (pre-transfer) at day 0 and in a Matrigel matrix (post-transfer) at day 1. Cells self-organize into 120 μm diameter spheroids at day 6 of culture forming a monolayer of cells surrounding a hollow lumen. The spheroids remain viable and lumenized at day 21. (**C**) Live/dead staining assay showing spheroid cell viability after 21 days in culture.

We used a previously developed Sacrificial Micromolding technique to culture geometrically and spatially controlled multicellular intestinal spheroids by allowing precise control over the initial size of the reconstituted cell aggregates^6^. Briefly, using simple photolithography and micromolding techniques, we fabricated agarose microwells that are 120 μm in diameter (Fig. 1A). Caco-2 cells were centrifuged into the sacrificial microwells and allowed to aggregate for 24 hours. The aggregates were then transferred to a Matrigel matrix in which they were cultured for 5 additional days. Matrigel was chosen as the ECM for its unparalleled imitation of the basement membrane *in vivo* as well as its ability to form stable but soft gels (<0.5 kPa) that match soft tissues like the small intestine^6^. These properties also allow Matrigel to promote cell morphogenesis as well as the diffusion of biochemical factors throughout the 3D matrix. We found that after 5 days in Matrigel, the Caco-2 cells self-organize into intestinal epithelial tissues with a confluent monolayer of cells surrounding a hollow lumen. Our previous study showed that the number of lumen formed is a function of the diameter of the cell aggregates^6^. We found that with an aggregate diameter of 120 μm, out of a total of 1072 spheroids, 1012 of the spheroids formed a lumen after 6 days in culture as determined by confocal microscopy (n experiment = 3; 477/504, 293/305, 242/263), yielding an average success rate of 94%. Additionally, we show that the spheroids are amenable to long-term culture. The spheroids remained stable and viable in Matrigel for 21 days. Figure 1B shows spheroids that were cultured for 6 days, 10 days, 14 days and 21 days in Matrigel. The spheroids maintained their structure and remained lumenized with a monolayer of cells surrounding the hollow lumen. Cell viability was confirmed by conducting a live/dead staining assay after 21 days in culture (Fig. 1C) and only 1 ± 0.3% of spheroids were determined to be dead using a propidium iodide stain. The ability of these spheroids to survive for 21 days in Matrigel increases the throughput of this system compared to the transwell system.

### Spheroids self-organize to form polarized tissues after 6 days in culture

**Figure 2:**
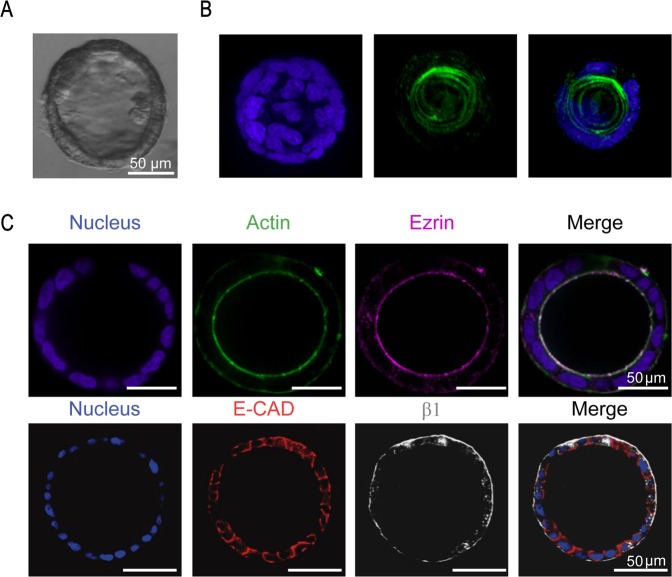
Lumenization and polarization of intestinal spheroids after 6 days in culture. (**A**) A representative brightfield confocal image of a lumenized intestinal spheroid at day 6. (**B**) 3D reconstruction of a confocal z-stack showing a representative spheroid forming a confluent monolayer (DAPI) and a continuous actin belt (phalloidin) surrounding the apical membrane. (**C**) Fluorescence images of a brush border shown by ezrin (purple) co-localized with actin (green) on the apical membrane facing the lumen (10X). E-cadherins (red) are forming between cells showing cellular cross-communication while β-1 integrin (grey) is expressed on the basolateral membrane engaging with the extracellular matrix (10X).

Polarization of epithelial cells is essential for maintaining their microenvironment and ensuring directional secretion of materials. We compared a small cell aggregate (35 μm in diameter) that did not lumenize to a lumenized 120 μm diameter spheroid. After 5 days in Matrigel, the lumenized spheroids formed a monolayer of cells surrounding a hollow lumen and displayed polarization with a continuous actin belt expressed on the apical membrane facing the lumen. The un-lumenized aggregates did not form a hollow lumen and instead displayed a cell-filled core, lacking the apical actin belt (Supplementary Fig. 2). Figure 2B shows a 3D reconstruction of a lumenized spheroid with a confluent cell monolayer and actin lining the perimeter of the apical membrane facing the lumen. The spheroids display brush border formation as shown by ezrin on the apical membrane (Fig. 2C). Additionally, β-1 integrin which receives and transduces signals from the ECM and is deeply involved in the epithelial polarization process is shown to be expressed on the basolateral side suggesting engagement with the ECM. E-cadherins are shown to be expressed between cells suggesting cross-communication between cells.

### Spheroids display fundamental intestinal barrier functions

**Figure 3:**
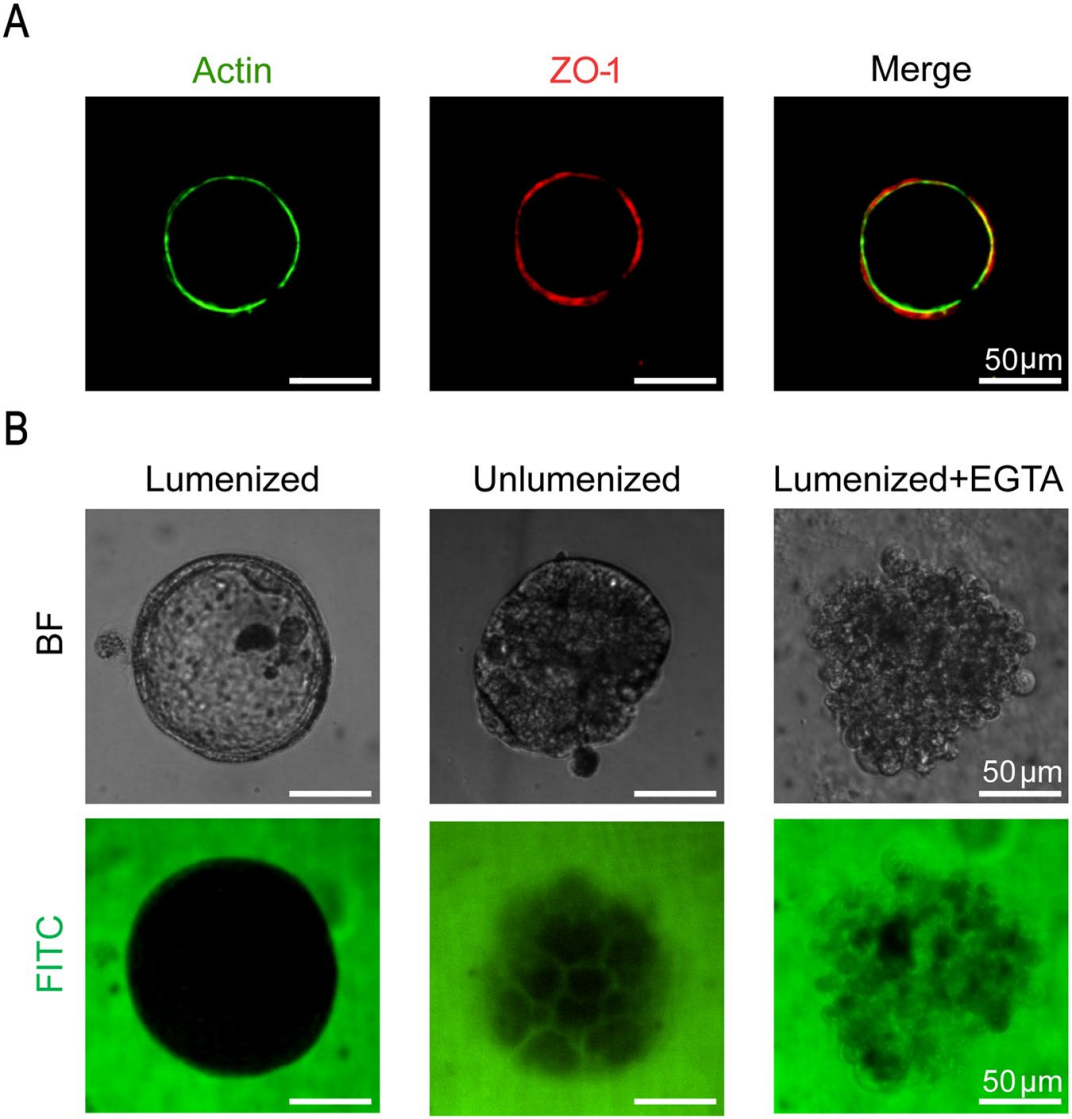
Barrier integrity of intestinal spheroids. (**A**) Fluorescent confocal image of a spheroid stained for tight junctional protein ZO-1 (red) expressed on the apical membrane and co-localized with actin (green). (**B**) Barrier integrity assay showing exclusion of 4 kDa FITC-dextran after incubation for 3 h in a lumenized spheroid while the FITC-dextran penetrates between the cells of an un-lumenized aggregate. Co-incubation of a lumenized spheroid with 16 mM EGTA chelates the tight junctions between the cells.

To test the barrier function of the spheroids, we first stained for zonula occludens (ZO-1) protein which is one of the essential proteins in the formation of the tight junctional complex in epithelial cells. This barrier limits the diffusion of molecules that are transported via the paracellular route across the small intestine^8^. We showed that the spheroids express ZO-1 co-localized with the actin on the apical membrane of the spheroids (Fig. 3A). We also observed that the surface of the intestinal spheroids displays low permeability to high molecular weight dextrans. 2 mM 4 kDa FITC-dextran was incubated on the basolateral side of lumenized spheroids and un-lumenized aggregates for 3 hours. Figure 3B shows that the FITC-dextran was mostly excluded from the lumen of spheroids suggesting a tight epithelial barrier, while the un-lumenized cell aggregate shows penetration of FITC-dextran between the cells. Barrier function of lumenized spheroids was immediately lost by co-incubation with 16 mM EGTA, which serves as a calcium chelating agent and thereby disrupts the tight junctions between the cells^9^. The disruption of tight junctions causes the spheroids to lose their lumenized structure and become leaky allowing for the penetration of FITC-dextran between cells.

**Figure 4:**
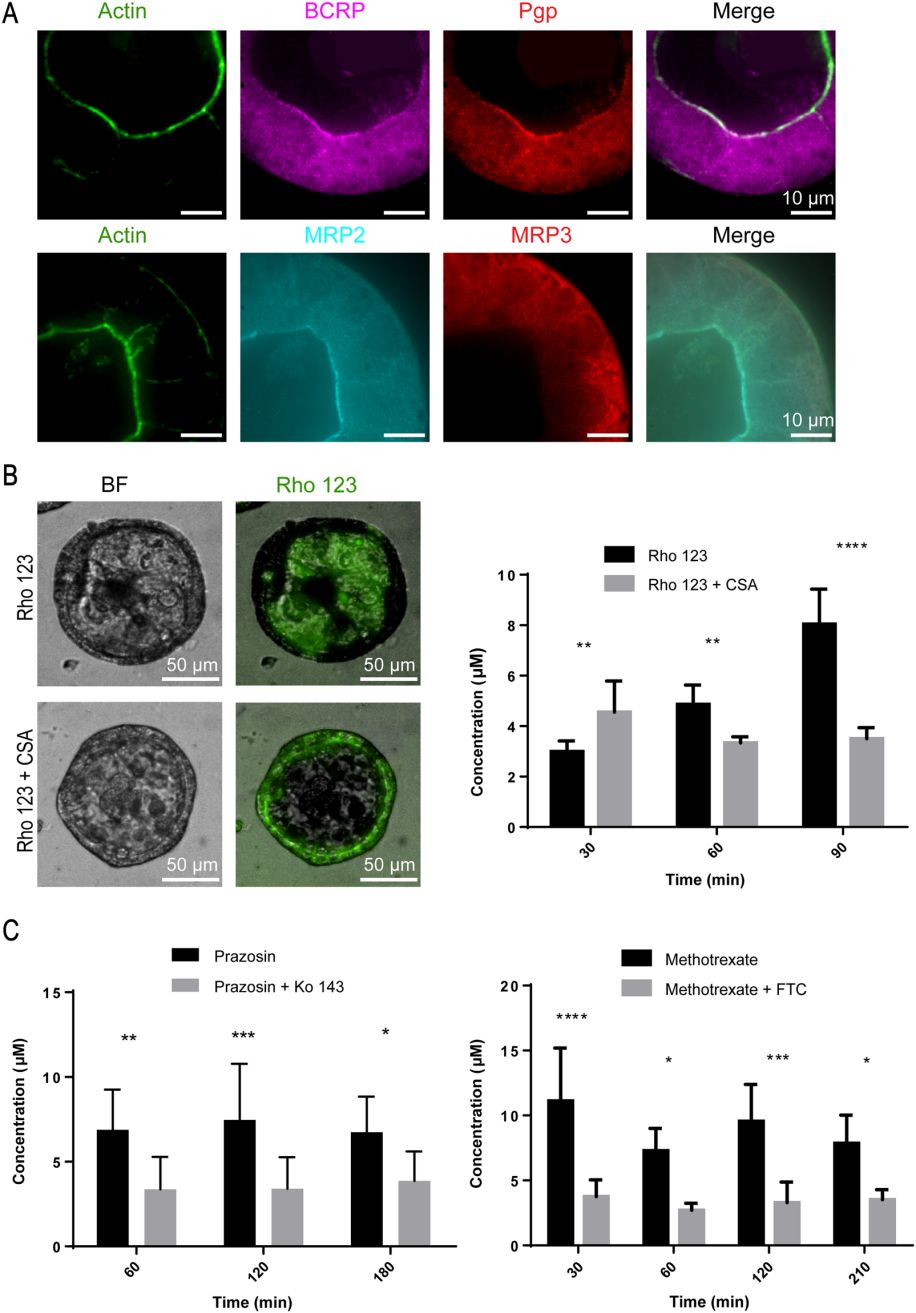
Transporter expression and function. (**A**) Immunofluorescence images showing expression of Pgp (red), BCRP (purple), and MRP2 (cyan) on the apical membrane facing the lumen and MRP3 (red) on the basolateral membrane (60X). (**B**) Fluorescence images showing Pgp model substrate, Rh 123, accumulating in the lumen of spheroids. Co-incubation with Pgp inhibitor CSA leads to accumulation of Rh 123 inside the cells. Bar graph depicts the influx of Rh 123 into the spheroid (black bars) while the luminal concentration is lower when Rh 123 is co-incubated with CSA (grey bars). The graph shows the mean Rh 123 concentration at different time points. (Mean ± SD; **p < 0.01; ****p < 0.0001, n = 10). (**C**) Bar graphs depicting the accumulation of BCRP substrates prazosin and methotrexate inside the lumen (black bars) while the luminal concentration is lower when the spheroids are co-incubated with the BCRP inhibitors Ko143 and fumitremorgin C (FTC), respectively (grey bars). (Mean ± SD; *p < 0.05; ***p < 0.001, n = 10). Statistical analyses were performed using two-way ANOVA and Sidak’s multiple comparison test.

**Figure 5:**
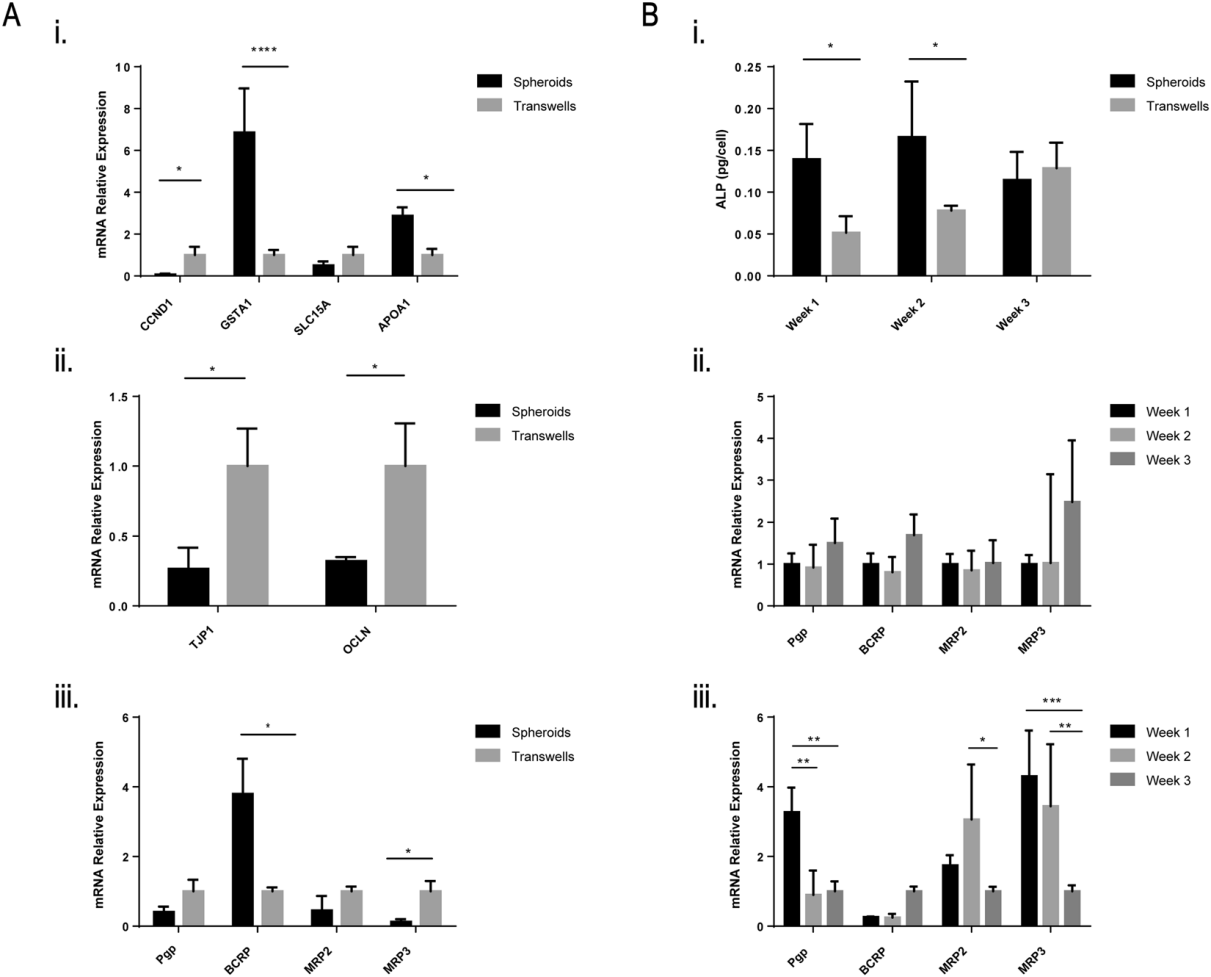
(**A**) Comparison between intestinal spheroids at day 6 in culture and monolayers on transwells after 3 weeks in culture showing (i) lower mRNA expression levels of proliferation marker CCND1 and higher expression of differentiation markers GSTA1 and APOA1 in spheroids compared to transwells, (ii) lower expression levels of tight junction proteins TJP1 and OCLN in spheroids, and (iii) more physiological transporter expression levels in spheroids. (**B**) Maturation of spheroids vs. transwells over time. (i) Activity of alkaline phosphatase is higher at week 1 and week 2 in spheroids compared to transwells, (ii) transporter expression remains constant over three weeks in spheroids compared to (iii) significant variations in expression levels in transwells over three weeks. (Mean ± SD; *p < 0.05; **p < 0.01; ****p < 0.0001; n = 3) Statistical analyses were performed using two-way ANOVA with Sidak’s or Tukey’s multiple comparisons tests.

Next, we examined the spheroids for efflux pump activity, which is responsible for the active transport of a variety of drugs out of the cells and back into the intestinal lumen, decreasing their oral bioavailability. First, we studied the expression of four highly abundant efflux transporters in the small intestine. P-glycoprotein (Pgp), Breast Cancer Resistance Protein (BCRP), and Multidrug Resistance-associated Protein 2 (MRP2) are responsible for limiting the intestinal absorption and oral bioavailability of many clinically important and frequently prescribed drugs such as immunosuppressants, antibiotics, anticancer drugs, HIV protease inhibitors, and cardiac drugs^10^. We showed that these three transporters are correctly localized on the apical membrane of the spheroids, while Multidrug Resistance-associated Protein 3 (MRP3) is correctly localized on the basolateral membrane (Fig. 4A). Table 1 includes antibodies used for immunohistochemistry. Furthermore, we measured mRNA expression levels of these four transporters after 6 days, 2 weeks, and 3 weeks and found no significant difference in expression levels (Fig. 5Bii). This suggests that the spheroids have reached a mature and differentiated state after 6 days in culture. Table 2 includes primer sequences used for assessing mRNA expression levels. As a proof-of-concept, to assess the function of efflux transporters across the spheroids, we used rhodamine 123 (Rh 123) as a model Pgp substrate. When delivered on the basolateral membrane, Rh 123 gets actively transported into the cells^11^. Pgp then actively pumps Rh 123 out of the cell and into the lumen. Upon delivery of 5 µM Rh 123 to the basolateral side of the spheroids, it rapidly accumulated in the apical lumen reaching a concentration of 8 µM within 90 minutes (Fig. 4B). However, upon treatment with 15 μM Cyclosporine A (CSA), a Pgp inhibitor, Rh123 accumulated inside the cells and was unable to be transported out into the lumen by Pgp. Previous studies have shown that Rh 123 partitions into the mitochondrial membranes of cells^12^ which explains the high fluorescence intensities detected inside the cells upon inhibition of Pgp.

**Table 1:** Antibodies and dilutions used for immunohistochemistry.

Antibody	Product	Dilution
Anti-rabbit Pgp	Abcam 103477	1:50
Anti-rabbit BCRP	Abcam 3380	1:50
Anti-Mouse Mrp2	Invitrogen MA-26536	1:50
Anti-rabbit MRP3	Abcam 107083	1:50
Anti-Mouse ZO-1	Invitrogen 339188	1:100
Anti-Rabbit Ezrin	Invitrogen 357300	1:50
Alexa 488 Phalloidin	Life Technologies 1726566	1:500
DAPI	Vector Laboratories H-1200	1:400

**Table 2:** Primer sequences used and the name and function of proteins they encode.

Gene	Primer Sequence	Protein encoded	Function/Localization
MDR1	F 5′-GCC AAA GCC AAA ATA TCA GC 3′ R 5′-TTC CAA TGT GTT CGG CAT 3′	P-glycoprotein (P-gp)	Efflux Transporter/Apical membrane
ABCG2	F 5′-TGC AAC ATG TAC TGG CGA AGA 3′ R 5′-TCT TCC ACA AGC CCC AGG 3′	Breast cancer resistance protein (BCRP)	Efflux Transporter/Apical membrane
ABCC2	F 5′-TGA GCA AGT TTG AAA CGC ACAT 3′ R 5′-AGC TCT TCT CCT GCC GTC TCT 3′	Multi-resistance protein 2 (MRP2)	Efflux Transporter/Apical membrane
ABCC3	F 5′-CAC CAA CTC AGT CAA ACG TGC 3′ R 5′-GCA AGA CCA TGA AAG CGA CTC 3′	Multi-resistance protein 2 (MRP3)	Efflux Transporter/Basal membrane
CCND1	F 5′-CAATGACCCCGCACGATTTC 3′ R-CATGGAGGGCGGATTGGAA 3′	Cyclin D1	Cell cycle progression in early G1 phase/cytoplasm
SLC15A	F 5′-TGTCCACCGCCATCTACCATA 3′ R 5′-CCACGAGTCGGCGATAAGAG 3′	Peptide transporter 1 (PEPT 1)	Uptake transporter responsible for the absorption of dietary di- and tripeptides from the small intestinal lumen/apical membrane
GSTA1	F 5′-AGCCGGGCTGACATTCATCT 3′ R 5′-TGGCCTCCATGACTGCGTTA 3′	Glutathione-transferase A 1	Detoxification of electrophilic compounds/ endoplasmic reticulum and outer mitochondrial membrane
APOA1	F 5′-CCAAAAAGCAGCTAGTGAAACC 3′ R 5′-AGTTGCAGTGCGGATGGAA 3′	Apolipoprotein A-I	Phospholipid transporter/apical membrane
TJP1	F 5′-ACC AGT AAG TCG TCC TGA TCC 3′ R 5′-TCG GCC AAA TCT TCT CAC TCC 3′	Zonula Occludin (ZO-1)	Tight Junction scaffolding protein/apical membrane
OCLN	F 5′-CGGG CGA GTC CTG TGA TGA G 3′ R 5′-TCT TGT ATT CCT GTA GGC CAG T 3′	Occludin	Tight Junction Protein/apical membrane
L19	F 5′-TCGCCTCTAGTGTCCTCCG 3′ R 5′-GCGGGCCAAGGTGTTTTTC 3′	Ribosomal protein	Housekeeping

Similarly, we studied the efflux function of BCRP using 5 μM prazosin and 10 μM methotrexate which are both substrates for BCRP. Upon treatment with the potent inhibitors Ko143 (50 µM) or FTC (2 µM), respectively, the luminal concentrations of both drugs decreased significantly (Fig. 5C). Taken together, these data suggest that the surface of the spheroids forms a tight barrier expressing functional efflux transporters that limit the absorption of foreign substrates into the spheroid.

### Spheroid platform is more robust and more physiological than 2D monolayers

Since the transwell system is the current gold standard model for intestinal absorption, we compared it to the intestinal spheroid system to characterize the role that the ECM and the 3D architecture play in instructing cellular differentiation and gene expression.

The Caco-2 cell line models the phenotypic changes epithelial cells undergo when they migrate along the crypt axis toward the luminal surface. Upon differentiation, Caco-2 cells decrease proliferation by down-regulating genes involved in cell cycle progression and DNA synthesis, while up-regulating genes involved in drug metabolism and transport^13^. We compared mRNA expression levels of intestinal epithelial differentiation and proliferation markers in the spheroids (grown for 6 days) and monolayers grown on transwell inserts for 21 days (Fig. 5Ai). Higher levels of CCND1, the gene encoding cyclin D1 protein, were measured in transwells compared to spheroids. CCND1 is responsible for cell cycle progression in early G1 phase and is a marker for cell proliferation. Inhibition of cyclin D1 in Caco-2 cells has been shown to inhibit cell proliferation and marks a more differentiated phenotype^14^. On the other hand, Glutathione-transferase A 1 (GSTA1) is a Phase II conjugating enzyme that functions in the detoxification of electrophilic compounds, including carcinogens, therapeutic drugs, environmental toxins and products of oxidative stress by conjugation with glutathione. We found the GSTA1 gene to be approximately 6 fold higher in spheroids than in transwells, indicating a more differentiated cell phenotype^15^. This is consistent with previous reports that have shown members of the glutathione S-transferase family to be among the highly induced genes during Caco-2 cell differentiation^13^. Additionally, higher production of apolipoproteins, which are responsible for binding and transport of lipids in the small intestine, is associated with a more differentiated cell phenotype that resembles small intestinal enterocytes more closely^16^. We measured a 2.9 fold increase in APOA1 expression in spheroids compared to transwells. APOA1 is the gene encoding apolipoprotein A-I, a component of high-density lipoprotein (HDL) which is responsible for transporting cholesterol and other phospholipids to the bloodstream. The increased expression of APOA1 again indicates a more differentiated phenotype in spheroids and is consistent with published observations regarding the regulation of apolipoprotein gene expression in enterocytes^16^.

Previous reports have shown that Caco-2 monolayers cultured on transwells for 21 days form a tighter barrier than the human small intestine because of their colonic origin^17^. We measured the gene expression levels of ZO-1 (TJP1) and occludin (OCLN) in the transwell model compared to the spheroids (Fig. 5Aii). We found that even though the spheroids are more differentiated and develop a more mature intestinal epithelial phenotype, the expression of ZO-1 was 80% lower and occludin was 60% lower than in transwells. In order to elucidate the role of the ECM in guiding changes in tight junction gene expression levels, we compared monolayers grown on Matrigel coated transwells (0.5 mg/ml Matrigel) to monolayers cultured on the traditional collagen coated transwells (Supplementary Fig. 3). Lower gene expression levels of ZO-1 and occludin were measured on Matrigel coated transwells similar to the spheroids suggesting that these changes might mainly be due to the effect of the ECM proteins.

On a biochemical level, brush border enzyme specific activity is commonly used as a marker for intestinal epithelial differentiation. For instance, studies have reported increases in the specific activities of brush border enzymes after culturing Caco-2 cells on different basement membrane proteins. These changes suggest that interactions with basement membrane proteins may promote intestinal epithelial differentiation^18^. The activity of alkaline phosphatase, a digestive enzyme on the apical brush border of intestinal enterocytes, was compared in spheroids and transwells over a period of 3 weeks (Fig. 5Bi). After 6 days in culture, activity levels were found to be 2.7 times higher in spheroids. After 14 days, activity levels show 2 fold higher levels in spheroids. No significant difference was detected at 21 days between the two models. These data suggest that when cultured in a 3D environment, Caco-2 cells reach a more mature and differentiated enterocyte-like phenotype over a shorter period of time compared to when cultured on transwells. This further highlights the advantage of using this model for drug screening.

Intestinal transporters play a crucial role in the oral absorption of a wide variety of drugs. Deviations in transporter expression levels between the Caco-2 monolayer model and the human jejunum could potentially result in inaccurate classification of the permeability and intestinal absorption of compounds, especially for drugs whose pharmacokinetics are heavily determined by active transport. We measured a 2.7 fold increase in expression of BCRP in spheroids compared to monolayers cultured on transwells for 21 days (Fig. 5Aiii). Furthermore, MRP3 was downregulated 7.8 fold in spheroids compared to transwells. These trends suggest a more differentiated phenotype that is physiologically more similar to the human jejunum in which BCRP has been shown to be expressed at 1.5–3 fold higher levels and MRP3 at 0.2–0.3 lower levels compared to monolayers on transwells^19,20^. In order to elucidate the role of the ECM in guiding changes in transporter gene expression levels, we again compared transporter expression levels of monolayers grown on Matrigel coated transwells and collagen coated transwells and found no significant difference in transporter gene expression levels. This suggests that these changes are mainly due to the 3D tissue-like architecture of the spheroids (Supplementary Fig. 3). Furthermore, while spheroid transporter levels did not vary significantly after they reached a mature state at day 6, the transporter expression levels varied from week 1 to week 3 on transwells (Fig. 5Bii,iii). This suggests that while Caco-2 cells require 3 weeks of culture on transwells to reach a mature state with a steady expression of transporters, it only takes 6 days for the spheroids to reach a more mature and more biomimetic state. This implies that spheroids will yield consistent and reproducible results if used in the timeframe between 6 days and 21 days in Matrigel.

This comparison between intestinal spheroids cultured using Sacrificial Micromolding and transwell monolayers highlights the advantages and the substantial potential of this intestinal spheroid platform in facilitating higher-throughput and more robust screening of oral drugs as well as studies of intestinal transport.

### Reversal of apical-basal polarity using Matrigel beads

Even though the mechanisms for establishing epithelial polarity are not well understood, it is widely recognized that signals from the ECM are essential for determining the basal membrane and establishing polarity. Additionally, epithelial cells show higher sensitivity to disturbances to apical-basal polarity in 3D culture than in 2D culture conditions^21^. Previously, we developed a technique to non-intrusively deliver microparticles to the lumen of spheroids without affecting the self-organization process^22^.

**Figure 6:**
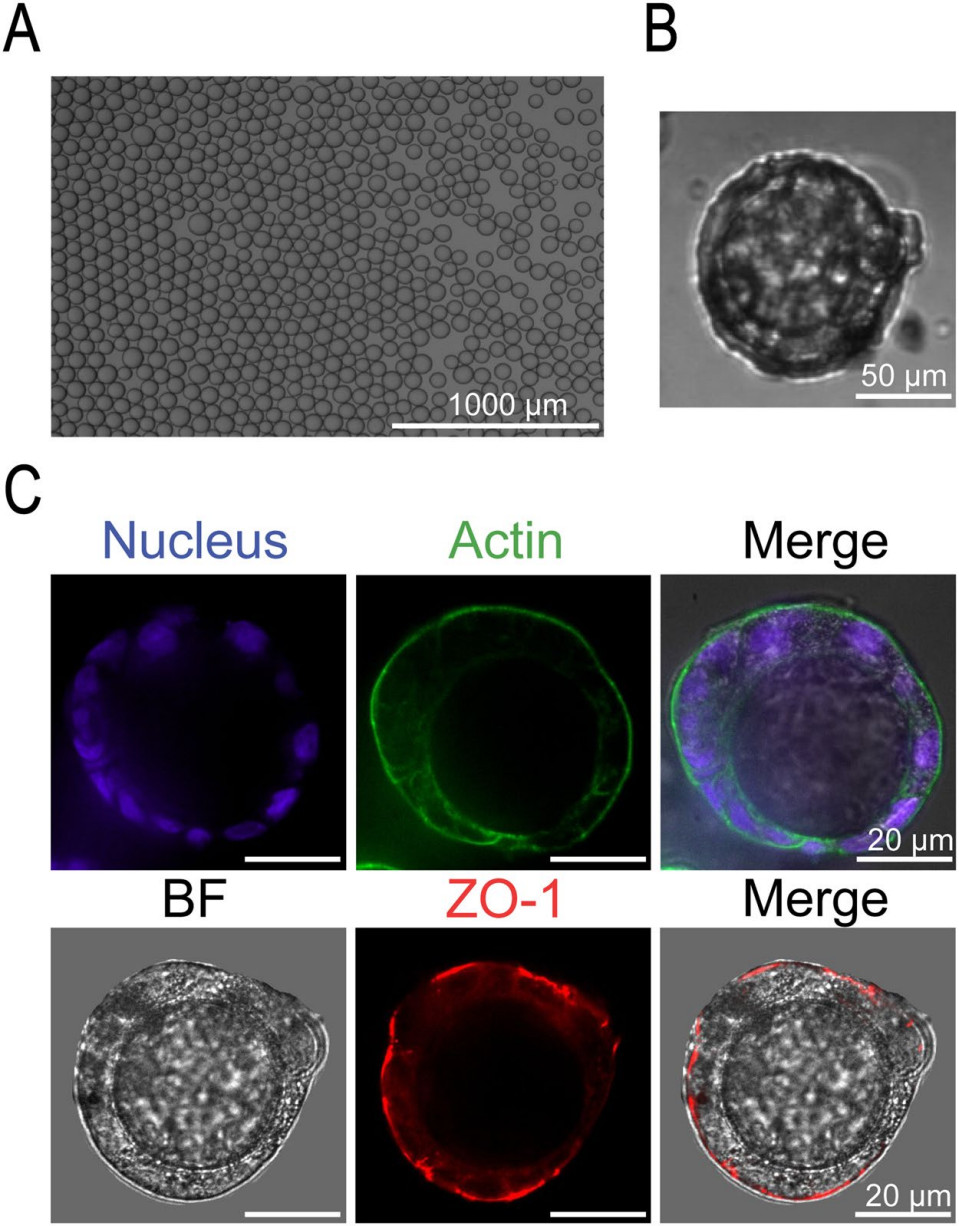
Reversal of apical-basal polarity using Matrigel beads. (**A**) Representative image of Matrigel beads in oil (30–60 μm diameter). (**B**) Brightfield image showing Matrigel bead surrounded by cells in an agarose microwell (10X). (**C**) Fluorescent images (40X) showing a continuous monolayer of cells surrounding a Matrigel bead shown by the DAPI stain (blue) and reversal of polarity with actin (green) and tight junction protein ZO-1 (red) expressed on the outer membrane away from the Matrigel bead.

We were interested in investigating whether we can culture cells around Matrigel beads using our previously described non-intrusive delivery technique to achieve spheroids with inverted polarity. We used a flow-focusing microfluidic dropmaker to generate 30–60 μm diameter Matrigel beads (Fig. 6A). We then pre-aggregated the Matrigel beads with Caco-2 cells in agarose microwells for 24 h and then transferred the cell-bead aggregates to 24-well low adhesion plates for 5 days. Figure 6B shows a Matrigel bead surrounded by cells in an agarose microwell. The cells surround the bead and adhere to the Matrigel forming a monolayer and tight junctions. Using immunostaining, we then showed that indeed the cells displayed an inverted polarity expressing actin on the outer membrane away from the Matrigel bead (Fig. 6C). Additionally, ZO-1 was co-localized with the actin and expressed on the outer membrane. Inverting the polarity of these spheroids while maintaining a differentiated cell phenotype would be useful in studies of drug transport by allowing access to the apical membrane. This system can be used to conduct further studies of drug transport as well as studies of epithelial polarity.

## Discussion

Previous research has demonstrated the usefulness of the Caco-2 cell line in predicting drug absorption due to its human origin and its advantageous differentiation into an enterocyte-like phenotype after 21 days on transwells. However, compared to *in vivo* Caco-2 monolayers grown on transwells have a less permeable passive paracellular route, different transporter expression levels, and lower brush border digestive enzyme activity levels. Additionally, in order to expedite the discovery of orally available drugs, methods to shorten the time to reach a fully differentiated confluent Caco-2 cell monolayer are highly desirable^23^. As has been previously shown, moving cells from a 2D to a 3D environment and restoring interactions with the ECM can induce dramatic effects on gene expression, differentiation, and metabolism and can, thus, more accurately model the *in vivo* phenotype^24^.

Here, we showed that Caco-2 spheroids cultured using Sacrificial Micromolding are able to reproduce *in vivo* intestinal epithelial properties and functions after only 6 days in culture. Moreover, a comparison between Caco-2 cells grown on transwells for 3 weeks and spheroids grown for 6 days in an ECM showed that while spheroids do form a tight barrier to high molecular weight dextrans, they display lower tight junction gene expression levels and more physiological transporter expression levels as well as digestive enzyme activity levels. These attributes highlight the spheroid model as a more biomimetic and reproducible intestinal model with potential in facilitating high-throughput screening of oral drugs.

Finally, we have also shown the potential to reverse the polarity of the spheroids to gain superficial access to the apical membrane. We believe that a more extensive characterization of this experimental system is necessary to characterize potential changes in cell phenotype. However, this technique may prove useful experimentally. For example, these spheroids can be incorporated in microfluidic chips to expose the apical membrane to flow to obtain a more physiological system. Organ-on-a-chip technologies have proven to be powerful tools for providing precise control over essential model components including fluid flow, mechanical support and chemical signals in a dynamically changing environment^25^. Previous studies have shown that growing Caco-2 cells within a microchannel of a Gut-on-a-Chip microdevice under constant flow in the presence of cyclic mechanical strain led to the formation of a morphology reminiscent of intestinal villi^26^.The Caco-2 cells differentiated into multiple intestinal cell types and displayed multiple physiological functions of normal human intestinal villi. Incorporation of flow and mechanical strain into this model would enhance its utility even further making it a powerful alternative *in vitro* model for studies on intestinal physiology as well as drug development.

## Methods

### Spheroid culture

The human colon adenocarcinoma cell line (Caco-2) was maintained in 2D cell cultures as previously described^27^. Caco-2 cells between passage 4 and 20 were used for all experiments. Cells were tested for mycoplasma contamination and found negative.

Caco-2 spheroids were cultured using Sacrificial Micromolding as previously described^6^. Briefly, Caco-2 cells were aggregated in agarose microwells for 24 h before transfer to a Matrigel gel in a glass-bottom 24 well plate in which aggregates were allowed to grow for 5 additional days. Media was refreshed every 2 days.

### Transwell culture

Caco-2 cells (25*${10}^{3}$ cells/well) were seeded on high density polycarbonate transwell inserts (24-well, 0.4 uM pore size) as previously described^27^. Cells were cultured for 21 days unless otherwise noted. For Matrigel coated transwells, a Matrigel concentration of 0.5 mg/ml was chosen as the highest concentration still enabling the formation of a 2D monolayer. Cells were cultured for 21 days and transepithelial electrical resistance (TEER) was monitored. Only wells with TEER greater than 350 Ω⋅cm^2^ were used for experiments.

### Live/Dead staining assay

The live/dead staining assay was conducted using a 2 mg/ml propidium iodide stock (Sigma-Aldrich Co. LLC, P4170) at a 1:50 dilution and a 5 mg/ml fluorescein diacetate stock (Sigma-Aldrich Co. LLC, C-7521) at a dilution of 1:625 in PBS. As a control or the dead stain, spheroids were fixed using 4% PFA and stained with the live/dead stain.

### Immunohistochemistry

Caco-2 spheroids were fixed as described previously^6^. Antibodies used for staining and the dilutions used for immunohistochemistry are included in Table 1.

Cells were imaged with a Yokagawa CSU22 spinning disk confocal microscope.

### Barrier integrity assay

Caco-2 spheroids and Tranwells were incubated in 2 mM 4KDa FITC Dextran (Sigma-Aldrich, St. Louis, MO, USA) or 2 mM 4 kDa FITC Dextran and 16 mM EGTA (Fisher Scientific, Hampton, NH, USA for 3 h. Fluorescence intensity in the lumen was measured while keeping laser power and exposure time constant.

### Real-time quantitative reverse transcription- polymerase chain reaction

RNA was collected from the 2D culture using the RNeasy Mini kit (Qiagen, Venlo, Netherlands). The 3D aggregates were first extracted from Matrigel by incubating with the Cell Recovery Solution (Corning, Midland, MI, USA) for 2 hours on ice to obtain isolated aggregates, and then RNA was collected with the QIAShredder (Qiagen, Venlo, Netherlands) and RNeasy Mini kit (Qiagen, Venlo, Netherlands). cDNA was generated from the RNA extract with iScript cDNA Synthesis Kit (Bio-Rad, Hercules, CA, USA). qPCR reactions were performed using the SYBR Green PCR Master Mix (Applied Biosystems, Foster City, CA, USA) using L19 as the reference control. Primers sequences used and the proteins they encode are provided in Table 2.

### Alkaline phosphatase detection

Alkaline phosphatase activity was detected with the SensoLyte pNPP ALP Colorimetric Assay Kit (AnaSpec, Fremont, CA, USA) after normalizing cell count with the CyQUANT Cell Proliferation Assay (Invitrogen, Carlsbad, CA, USA). Briefly, cell extracts were collected by incubating either 2D cultures or extracted 3D aggregates in ALP assay buffer with 0.2% Triton-X for 10 minutes at 4 °C, then the solution was spun down at 2500 g for 10 minutes and the supernatant was collected as cell extracts. DNA content in the cell extracts were measured with the CyQUANT assay kit to estimate cell numbers in each sample. Reaction volume for each sample was adjusted so that an equal number of cells from each sample was assayed for ALP activity.

### Functional efflux assay

Caco-2 spheroids were serum starved for half an hour in serum free EMEM before conducting the transport experiments. Spheroids were incubated with either 5uM rhodamine 123 (ThermoFisher Scientific, Hampton, NH, USA) or BODIPY FL prazosin (Life Technologies, Carlsbad, CA, USA) or fluorescein methotrexate (Life Technologies, Carlsbad, CA, USA) in serum free EMEM. Inhibition studies were performed by first incubating the spheroids with 15 uM Cyclosporine A (Sigma-Aldrich, St. Louis, MO, USA) or 50 uM Ko 143 (Abcam, Cambridge, UK) or 2 μM fumitremorgin C (Abcam, Cambridge, UK) for 30 minutes at 37 °C. After removing the inhibitor solution the spheroids were then incubated with both substrate and inhibitor for 90 minutes. Spheroids were imaged at different time points using a confocal microscope. Fluorescence intensities were measured in the lumen of spheroids while keeping laser power and exposure time constant. Calibration curves of fluorescence intensities vs. drug concentration were used to calculate concentration inside the lumen. All experiments were conducted by taking the average of n = 10 spheroids per condition.

### Culture of Caco-2 cells around Matrigel beads

Matrigel beads were generated using a flow-focusing microfluidic dropmaker with a 40 μm × 40 μm dropmaking junction. A master mold for the device was fabricated with a height of 40 μm using a standard soft lithography procedure as previously described^28^ and casted in polydimethylsiloxane (Sylgard 184, Dow Corning, Midland, MI, USA). To generate droplets, syringe pumps (catalog no. NE-501, New Era, Buffalo, NY, USA) were operated at flow rates of 100 μL/h for a solution of 10 mg/mL Matrigel in water (Corning, CB40230, Midland, MI, USA) and 200 μL/h for fluorinated oil (3 M, HFE-7500) containing 2% (w/w) of a PEG-PFPE block copolymer surfactant (Ran Technologies, 008-Fluoro-surfactant, Houston, TX, USA). To prevent premature gelation of the Matrigel, a laboratory-grade nitrile glove was filled with ice water and secured to the Matrigel syringe to keep the solution at 4–6 °C. Following collection, Matrigel droplets were incubated 8 h at room temperature to allow complete gelation of the Matrigel phase.

Matrigel beads were washed by removing the bottom oil layer. 400 μl of 10% perfluoroctane (PFO) in HFE oil were added to the Matrigel layer and mixed gently. The mixture was transferred to a sieve and washed with more 10% PFO until the emulsions were broken. 2% BSA was added to the Matrigel beads and transferred to a centrifuge tube. A mixture of Caco-2 cells and Matrigel beads was centrifuged into 120 μm agarose microwells at a ratio of 1:10 beads to cells as described previously^22^. The beads and cells were aggregated in the agarose microwells for 24 hours and then transferred to 24-well low adhesion plates for 5 days. To prepare for staining the cell-bead aggregates were transferred to centrifuge tubes and centrifuged at a speed of 300 rpm. The supernatant was removed and the pellet was fixed and stained as described previously.

## Supplementary information

Supplementary Information

## Data Availability

All data generated or analyzed during this study are included in this published article (and its Supplementary Information files).
