# Supplementary material for: Human intestinal spheroids cultured using Sacrificial Micromolding as a model system for studying drug transport
Source: Sci Rep. 2019 Jul 9;9:9936. doi: 10.1038/s41598-019-46408-0 (PMC6616551; doi:10.1038/s41598-019-46408-0)
Supplement: Supplementary file 1 — Supplementary Information [file 41598_2019_46408_MOESM1_ESM.pdf]

Human intestinal spheroids cultured using Sacrificial Micromolding as a model system for studying drug transport

Karen E. Samy[a,b], Elizabeth S. Levy[a], Kiet Phong[a,b], Benjamin Demaree[a,b], Adam R. Abate[a,c], Tejal A. Desai[a,*]

[a]Department of Bioengineering and Therapeutic Sciences, University of California, San Francisco, CA, USA

[b]UC Berkeley – UCSF Graduate Program in Bioengineering, UCSF Mission Bay Campus, San Francisco, CA, USA

[c]Chan Zuckerberg Biohub, San Francisco, CA, USA

*Corresponding author

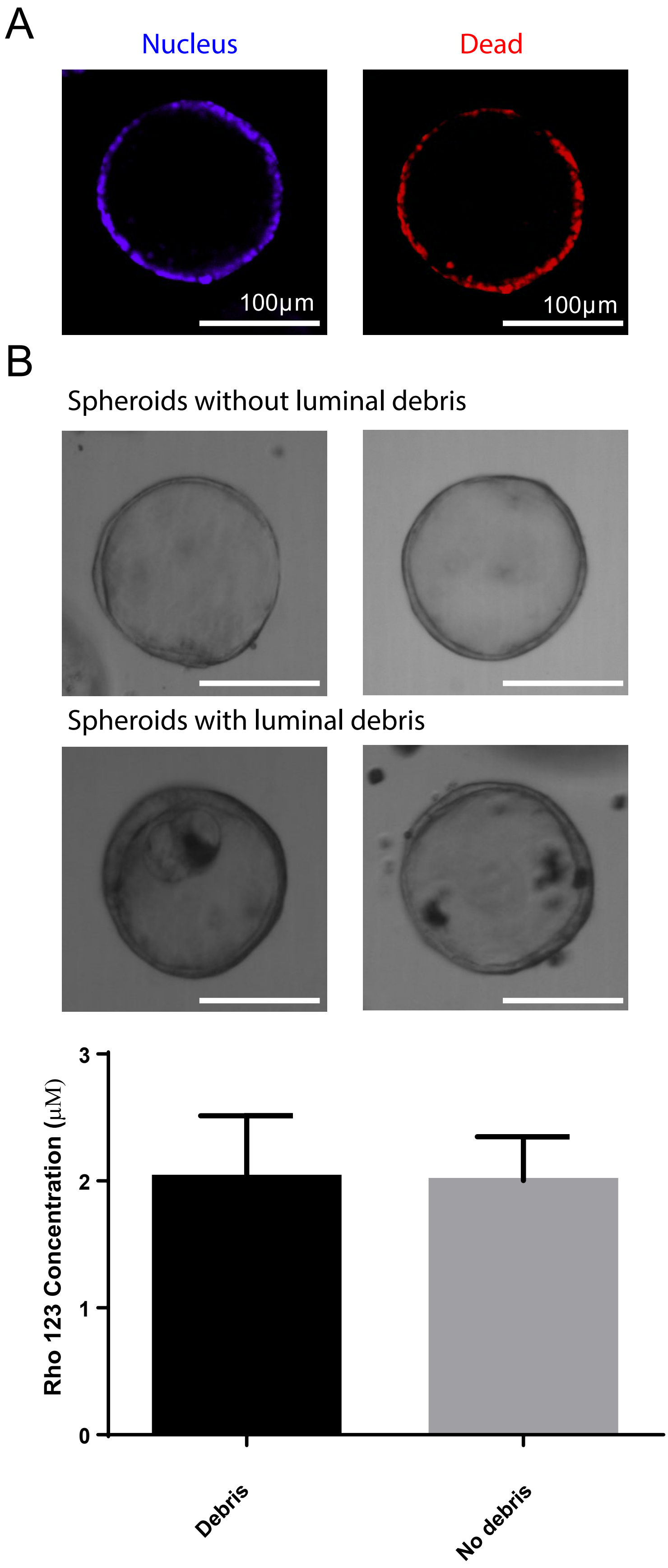

Supplementary Figure 1. The luminal space contains no dead cells and cell debris found in the lumen of some spheroids does not influence the functional fluorescence studies. A) Spheroids cultured for 21 days were fixed to kill the cells. The dead spheroids stained with a PI stain show a monolayer of dead cells surrounding a hollow lumen. No dead cells are found in the lumen. B) Brightfield representative images of spheroids with and without debris (Scale bar = 100 µm) and Rhodamine 123 luminal concentration measured after 30 minutes in spheroids with and without debris showing no significant difference in concentration (Mean ± SD; n=4). Statistical analyses were performed using two-way ANOVA with Sidak's or Tukey's multiple comparison tests.

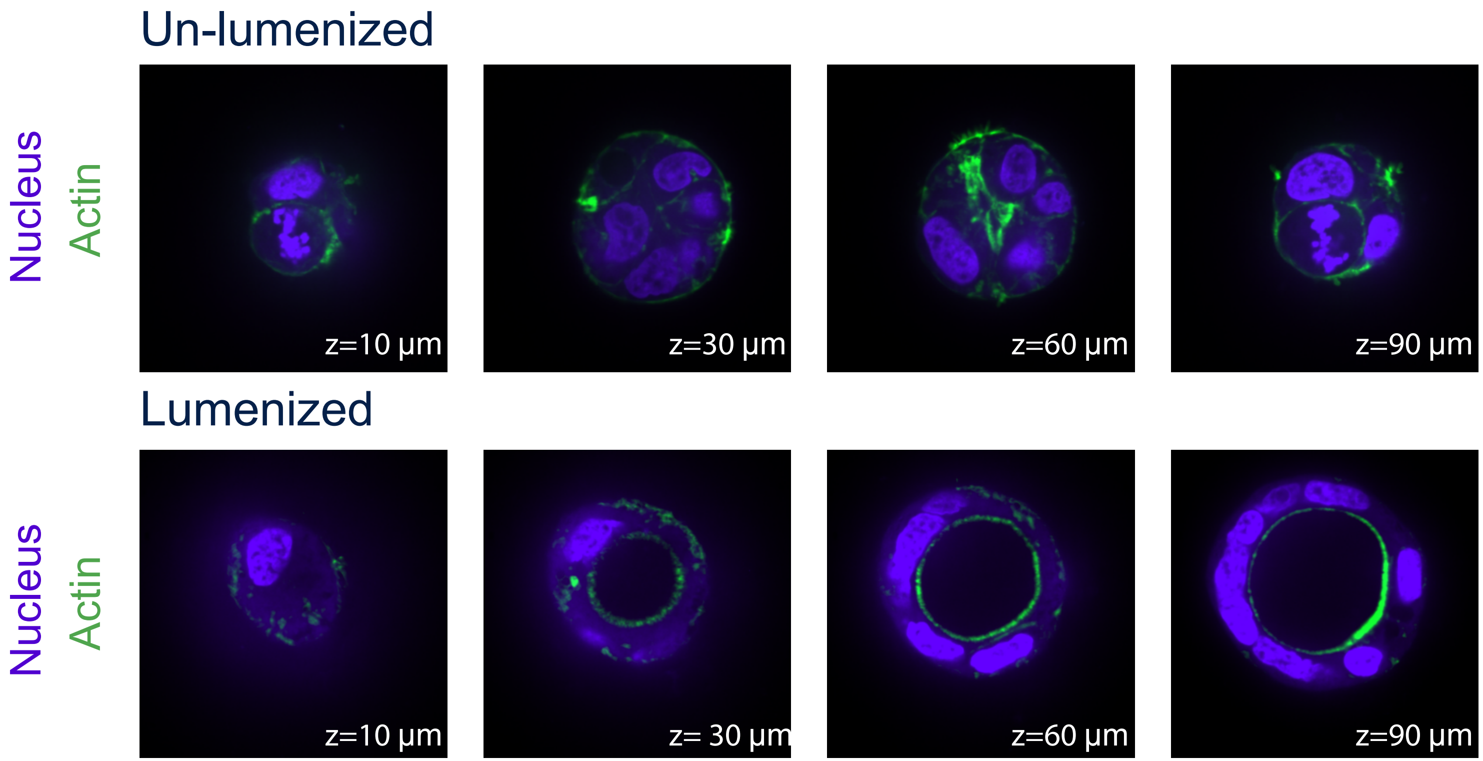

Supplementary Figure 2. Z-stack of lumenized spheroids and un-lumenized aggregates stained with DAPI (blue) and phalloidin (green). Lumenized spheroids display a continuous monolayer of cells surrounding a hollow lumen with an actin belt around the apical membrane whereas un-lumenized aggregates display a cell-filled core, lacking the apical actin belt.

A

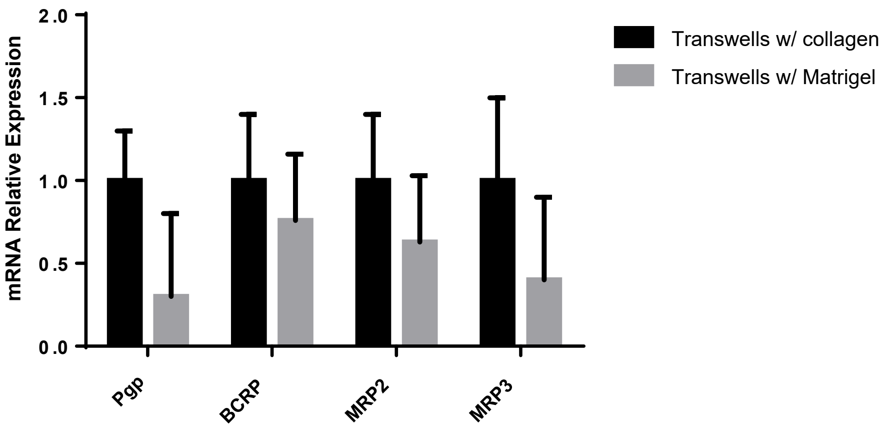

B

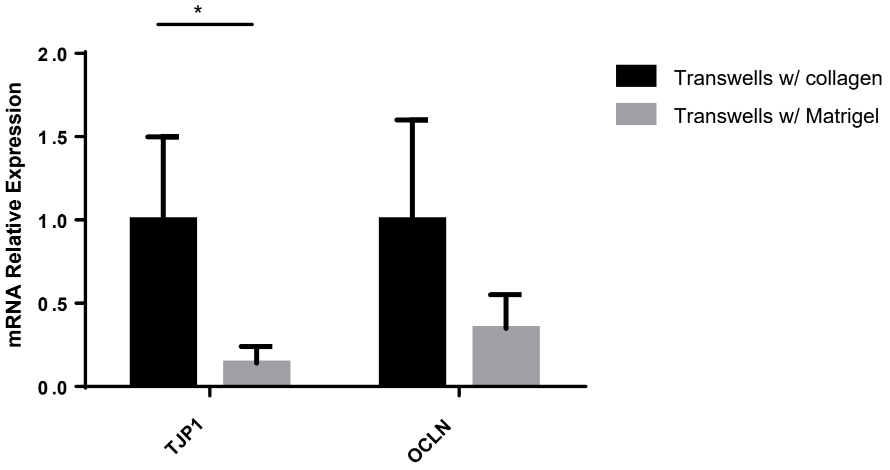

Supplementary Figure 3. Gene expression differences in transporter expression between spheroids and transwells are mainly due to the 3D architecture. A) Comparison between 2D Caco-2 monolayers grown on collagen coated transwells for 3 weeks and monolayers grown on Matrigel coated transwells for 3 weeks showing no significant difference in transporter expression levels. B) Downregulation of ZO-1 and occludin in Matrigel coated transwell monolayers compared to collagen coated monolayers suggesting an important role of ECM protein interactions. (Mean ± SD; *p<0.05; n=3) Statistical analyses were performed using two-way ANOVA with Sidak's or Tukey's multiple comparisons tests.
